# Complete genome sequence of *Paraburkholderia terrae* strain KU-46, a 2,4-dinitrophenol-degrading bacterium

**DOI:** 10.1128/mra.00282-24

**Published:** 2024-05-03

**Authors:** Tomoki Tanaka, Kenji Okano, Hiroaki Iwaki

**Affiliations:** 1 Department of Life Science and Biotechnology, Kansai University, Suita, Osaka, Japan; University of Southern California, Los Angeles, California, USA

**Keywords:** *Paraburkholderia*, 2,4-dinitrophenol, 4-nitrophenol, degrading bacterium, complete genome

## Abstract

*Paraburkholderia terrae* strain KU-46 has been studied for its capability to degrade 2,4-dinitrophenol. Here, we present the complete 10,833,180bp genome of this microorganism, comprising five circular chromosomes housing 9,797 protein-coding sequences. The genes responsible for 2,4-dinitrophenol and 4-nitrophenol degradation are located on chromosome 2.

## ANNOUNCEMENT

Strain KU-46 was isolated in 2003 by our laboratory from a surface soil sample collected from cropland in Suita, Osaka, Japan, based on its ability to grow using 2,4-dinitrophenol as the sole carbon and nitrogen source (1). This strain is unique in its ability to degrade 2,4-dinitrophenol via the 4-nitrophenol pathway (1, 2). Strain KU-46 was identified as *Paraburkholderia* (formerly *Burkholderia*) sp. based on the 16S rRNA gene sequence (AB293987) as well as biochemical characteristics (1). Members of the genus *Paraburkholderia* degrade various aromatic and aliphatic compounds (3–6). Here, we report the complete genome sequence of strain KU-46, which expands our understanding of the potentially useful bacterial genus *Paraburkholderia*.

Strain KU-46 was inoculated from our laboratory cryostock, and genomic DNA was isolated from cells after 18h of culture in 1/2 concentration of Miller’s Luria-Bertani (LB) medium (Merck) at 30°C using Wilson’s procedure (7) with some modifications (6). For PacBio sequencing, 4µg of genomic DNA underwent shearing to obtain fragments of the desired size using the Megaruptor 3 (Diagenode). Fragments ranging in sizes from 7 to 12 kb were then purified using AMPure PB magnetic beads (Pacific Biosciences). A 7–12-kb SMRTbell template library was prepared and sequenced using a PacBio Sequel IIe (Pacific Biosciences). A 10-µL library was prepared using PacBio SMRTbell Prep Kit 3.0 (Pacific Biosciences). The SMRTbell templates were annealed using the Sequel II Bind Kit 3.2 (Pacific Biosciences). Sequencing was conducted by Macrogen on the PacBio Sequel IIe platform using the Sequel II Sequencing Kit 2.0 and SMRT cell 8M Tray. To obtain high-quality data, adapter sequences were trimmed from the polymerase reads, and 99% accuracy or higher consensus sequences, referred to as High Fidelity (HiFi) reads (85,990 HiFi reads; 909,315,361 HiFi bases; *N*_50_ value of 11,914bp; mean length of 10,574bp; mean read quality of Q37), were generated by merging adapter trimmed reads yielded from a same Zero Mode Waveguide. The reads were *de novo* assembled using the Microbial Assembly application in SMRTlink v11.1.0.166339. The assembled genome was 10,833,180bp long and consisted of five circular replicons (Table 1). Genome sequence was annotated using DFAST (https://dfast.nig.ac.jp) (8). Default parameters were used for all tools unless otherwise noted.

**TABLE 1:** General genomic features of *Paraburkholderia terrae* KU-46

Genome	Length (bp)	GC content (%)	Depth	No. of coding sequences	No. of rRNAs	No. of tRNAs	Accession no.
Chromosome 1	3,660,500	62.8	81.9	3,179	12	58	AP029613
Chromosome 2	3,017,103	62.2	83.1	2,724	6	7	AP029614
Chromosome 3	2,148,366	62.0	82.3	1,889	0	2	AP029615
Chromosome 4	1,161,355	59.6	91.2	1,201	0	1	AP029616
Chromosome 5	845,856	59.8	88.8	804	3	3	AP029617
Total	10,833,180	61.9	83.9	9,797	21	71	

Strain KU-46 was re-identified as *Paraburkholderia terrae* through whole-genome comparisons using the Type (Strain) Genome Server (http://tygs.dsmz.de/) (9) with a digital DNA-DNA hybridization (formula *d*_4_) value of 70.6%. The genes responsible for 2,4-dinitrophenol degradation (2), *dnpAB* (locus_tag: PTKU46_42420 and PTKU46_42410), *dnpC1C2DFER* (locus_tag: PTKU46_42400 to PTKU46_42350), and *hqdA1A2BDC* (locus_tag: PTKU46_50570 to PTKU46_50530), are all located on chromosome 2. This complete genome sequence provides the genetic foundation for understanding the detailed catabolic mechanisms of nitrophenols in this specialized bacterium. It also helps shed light on the acquisition of the ability to degrade a wide variety of substances by bacteria of this genus.

## Data Availability

The genome sequence of strain KU-46 is available from DDBJ/EMBL/GenBank with accession numbers AP029613, AP029614, AP029615, AP029616, and AP029617. The associated BioProject, BioSample, and Sequence Read Archive accession numbers are PRJDB17035, SAMD00657987, and DRR513694, respectively.
